# Cost-Effectiveness Analysis of Six Immunotherapy-Based Regimens and Sunitinib in Metastatic Renal Cell Carcinoma: A Public Payer Perspective

**DOI:** 10.1200/OP.22.00447

**Published:** 2023-01-04

**Authors:** Minkyoung Yoo, Richard E. Nelson, Zachary Cutshall, Maura Dougherty, Manish Kohli

**Affiliations:** ^1^ Division of Epidemiology, Department of Internal Medicine, University of Utah School of Medicine, Salt Lake City, UT; ^2^ Informatics Decision Enhancement and Surveillance (IDEAS) Center, VA Salt Lake City Healthcare System, Salt Lake City, UT; ^3^ University of Utah School of Medicine, Salt Lake City, UT; ^4^ Department of Economics, University of Utah, Salt Lake City, UT; ^5^ Division of Oncology, Department of Medicine, University of Utah, Salt Lake City, UT; ^6^ Huntsman Cancer Institute, Salt Lake City, UT

## Abstract

Several new treatment combinations have been approved in metastatic renal cell carcinoma (mRCC). To determine the optimal therapy on the basis of cost and health outcomes, we performed a cost-effectiveness analysis of approved immunotherapy-tyrosine kinase inhibitor/immunotherapy drug combinations and sunitinib using public payer acquisition costs in the United States.

**METHODS:**

We constructed a decision model with a 10-year time horizon. The seven treatment drug strategies included atezolizumab + bevacizumab, avelumab + axitinib, pembrolizumab + axitinib, nivolumab + ipilimumab (NI), nivolumab + cabozantinib, lenvatinib + pembrolizumab, and sunitinib. The effectiveness outcome in our model was quality-adjusted life-years (QALYs) with utility values on the basis of the published literature. Costs included drug acquisition costs and costs for management of grade 3-4 drug-related adverse events. We used a partitioned survival model in which patients with mRCC transitioned between three health states (progression-free, progressive disease, and death) at monthly intervals on the basis of parametric survival function estimated from published survival curves. To determine cost-effectiveness, we constructed incremental cost-effectiveness ratios (ICERs) by dividing the difference in cost by the difference in effectiveness between nondominated treatments.

**RESULTS:**

The least expensive treatment was sunitinib ($357,948 US dollars [USD]-$656,100 USD), whereas the most expensive was either lenvatinib + pembrolizumab or pembrolizumab + axitinib ($959,302 USD-$1,403,671 USD). NI yielded the most QALYs (3.6), whereas avelumab + axitinib yielded the least (2.5). NI had an incremental ICER of $297,465 USD-$348,516 USD compared with sunitinib. In sensitivity analyses, this ICER fell below $150,000 USD/QALY if the initial 4-month cost of NI decreased by 22%-38%.

**CONCLUSION:**

NI was the most effective combination for mRCC, but at a willingness-to-pay threshold of $150,000 USD/QALY, sunitinib was the most cost-effective approach.

## INTRODUCTION

The estimated incidence of renal cell cancer in the United States will be 79,000 with an estimated 13,900 deaths in 2022.^1^ Significant advances have been made in prolongation of longevity of life since 2017 with several novel drug combinations including immunotherapies that have been approved for the management of metastatic renal cell cancer (mRCC). These novel immunotherapy (IO)-based drug combinations directed against the programmed cell death (programmed cell death protein 1-programmed death-ligand 1) receptor axis and also combined with vascular endothelial growth factor receptor-tyrosine kinase inhibitors (TKI) or with other IO drugs (immunotherapy-immunotherapy [IO-IO]) were compared with single-agent sunitinib, a vascular endothelial growth factor receptor-TKI first approved for treating mRCC in 2006, and demonstrated significantly improved response rates, progression-free survival (PFS), and overall survival (OS).^2^

However, a significant proportion of patients receiving these novel drug combinations have treatment-related adverse events (trAEs) and/or immune-related adverse events (irAEs). In one systematic review, the pooled incidence of adverse events in patients with mRCC undergoing contemporary drug treatments with IO and IO-TKI combinations was 88.3% for any-grade trAEs, 42.7% for any-grade irAEs, 36.1% for grade ≥ 3 trAEs, and 11.1% for grade ≥ 3 irAEs, whereas the overall mortality from drug-related toxicity was 0.9%.^3^ Costs of management of treatment toxicities can include outpatient management of rash and hypo- and hyperthyroidism to inpatient hospitalizations secondary to pneumonitis, colitis, and diarrhea. Management of these drug combinations can also include a 14% chance of having to deliver high-dose corticosteroids.^3^ These increased management costs of drug delivery and the impact on quality of life of patients undergoing first-line IO-TKIs or IO-IO combinations for mRCC should be weighed against the demonstrated gains in survival when considering the most optimal cost-effective treatment choice.

We conducted a cost-effectiveness analysis (CEA) of five recent drug combination approvals in the management of mRCC in the first-line setting from randomized clinical trials and a sixth first-line treatment regimen (atezolizumab + bevacizumab [AB], which has demonstrated increased progression-free survival interval but for which survival benefit is pending further follow-up). These included nivolumab + ipilimumab (NI; CHECKMATE-214 trial),^4^ pembrolizumab + axitinib (PA; KEYNOTE-426 trial),^5^ avelumab + axitinib (AA; JAVELIN RENAL 101 trial),^6^ nivolumab + cabozantinib (NC; CHECKMATE 9 ER trial),^7^ lenvatinib + pembrolizumab (LP; CLEAR trial),^8^ and AB (IMmotion-151 trial).^9^ Since all the above novel drug combinations were compared with sunitinib in these trials, sunitinib was used as a reference for conducting the CEA (Data Supplement, online only).

## METHODS

### Study Design Overview

We developed a decision analytic model to describe the treatment course of patients diagnosed with mRCC using costs from the perspective of two US public payers: the Department of Veterans Affairs (VA) and the Centers for Medicare & Medicaid Services (CMS). We estimated the cost and effectiveness of the seven first-line treatments for mRCC: (1) AB, (2) AA, (3) PA, (4) NI, (5) NC, (6) LP, and (7) sunitinib. The effectiveness measure was quality-adjusted life-years (QALYs), a metric that encompasses both duration and quality of life. Costs included the direct costs of the drug and administration and a set of drug adverse events (ADEs) for each treatment. Each model cycle represented 4 weeks, and the time horizon of the study was 10 years, as the life expectancy of patients with mRCC rarely crosses 10 years. We applied a half-cycle correction and a 3% annual discount rate for both costs and effectiveness outcomes. The model was programmed in TreeAge Pro 2018 (TreeAge Software, Williamstown, MA), and statistical analyses on survival functions were performed in R (version 4.1.1, RStudio version 1.4.1717).

### Model Structure

**FIG 1.**
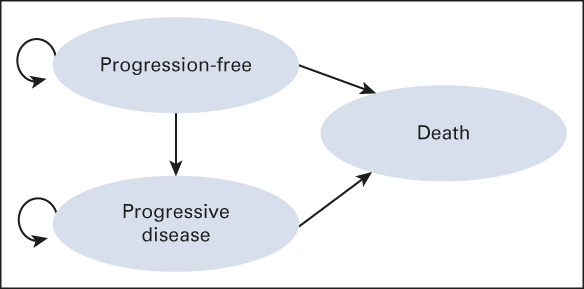
Health states with arrows depicting allowable transitions.

Our decision model was constructed to perform a partitioned survival analysis (PSA), a commonly used approach for CEA studies in oncology.^10-12^ The model included three health states: progression-free, progression, and death (Fig 1). The PSA derived the proportion of patients in each health state at each point in time using the PFS and OS curves for the intention-to-treat population^12^ from each published clinical trial. We assumed that patients' first-line treatments continued until disease progression or for 2 years during the 10-year time period. This assumption for length of treatment with first-line regimens in patients who continue to derive benefit was made in line with best clinical practice standards in the community and can be variable as there are no clear definitions proposed for the length of treatments in this clinical scenario. On progression of the disease or at the end of treatments in patients continuing to respond at the 2-year treatment period, we assumed that patients in all first-line treatment arms receiving IO-IO or IO-TKI combinations would receive subsequent treatment with sunitinib as second-line therapy until death, which is a listed subsequent line therapy option in National Comprehensive Cancer Network guidelines and in practice until recently. However, more recently, several second-line regimens have emerged and clinical practice for using these and sunitinib may vary. We also ran a model with the assumption that only 30% of patients would receive this second-line therapy on the basis of published trials and previous CEA studies.^13-15^

### Drug Effectiveness: Model Survival and Progression Risk Estimates

To extract the probability coordinates from the published Kaplan-Meier PFS and OS curves in the trials, we used Engauge Digitizer (version 12.1)^16^ and WebPlotDigitizer (version 4.5)^17^ software. To extrapolate mortality data to a 10-year time horizon, we applied an algorithm provided by Hoyle and Henley^18^ and generated pseudoindividual patient events and censoring data. Parametric survival functions for both PFS and OS were chosen on the basis of goodness-of-fit statistics (Akaike information criterion) among potential distributions: exponential, Weibull, logistic, lognormal, and log-logistic. In our study, the survival functions followed exponential, log-logistic, or Weibull distributions (Data Supplement). Since each of the six trials for new drugs included sunitinib as the comparator, we had six different sunitinib median PFS periods reported in the study-specific Kaplan-Meier curves. We ran our model separately using the highest (CheckMate 214 [NI *v* sunitinib]) and lowest (Javelin Renal 101 [AA *v* sunitinib]) reported median PFS among the six trials.

### Drug Toxicities

We included ADEs that were either grade 3 or 4 and were reported for at least 5% of patients for each first-line treatment from each trial. Treatment costs for each ADE were derived from Healthcare Cost & Utilization Project and other sources^19^ and were applied in the first cycle of the model. Additional treatment cost of ADEs for sunitinib was applied for those who received the subsequent treatment. The list of ADEs and their likelihood and treatment costs for each first-line treatment are summarized in the Data Supplement.

### Utility Inputs

QALYs were calculated in the model by weighting the time spent in each health state by health-related quality of life, as measured by a utility score, which ranges from 0 (death) to 1 (perfect health). Utility scores for each first-line treatment were derived from previously published data.^15,20-22^ Among IO combinations, the PFS utility values ranged from 0.87 for NC and LP on the basis of their efficacy in terms of median PFS^15^ to 0.77 for PA,^20^ whereas the utility score for sunitinib was 0.73^21^ (Data Supplement). The utility score for the progression health state was estimated at 0.1 less than the utility score for progression-free state for each first-line treatment.^22^

### Direct Drug Cost

We included drug costs of the first-line treatments from two different sources representing different US public payer perspectives: VA and CMS. For the VA perspective, we used the Federal Supply Schedule (FSS) pharmaceutical pricing data, which reflect negotiated prices between drug companies and VA, which is one of the largest purchasers of pharmaceuticals in the United States. For the CMS perspective, the direct Medicare/Medicaid drug costs were derived from the Medicare Part B Drug Average Sales Price (ASP) reported from the drug manufacturers without a Medicaid drug rebate agreement and the National Average Drug Acquisition Cost (NADAC) when ASP was not available. The ASP or NADAC was available for atezolizumab, avelumab, pembrolizumab, nivolumab, bevacizumab, and sunitinib. For the remaining drugs (axitinib, ipilimumab, cabozantinib, and lenvatinib), we calculated the CMS drug cost by multiplying the FSSTable cost by the ratio of CMS to FSS cost from the drugs for which the CMS cost was available. All direct drug costs were calculated on the basis of 71.4-kg patient.^20,23^ The direct costs and dosages of each drug for the seven first-line treatment are summarized in the Data Supplement. Average administration costs of $74.40 US dollars (USD; Current Procedural Terminology 96417) and $153.60 USD (Current Procedural Terminology 96413), derived from the CMS physician fee schedule, were added to the total costs for drugs that require injection administration. Per-cycle per-treatment cost estimates are summarized in the Data Supplement.

### Monitoring, Postprogression, and End-of-Life Costs

Monitoring, post-progression, and end-of-life costs were based on the secondary literature.^24^ Monitoring costs included monthly general practitioner visit and blood test and biannual computed tomography scans, and costs for best supportive care postprogression included general practitioner visits, community nurse visits, pain medications, and postdiscontinuation treatments. We assigned monitoring costs for all patients who underwent any treatment options whereas postprogression costs for patients who had progression of the disease or were at the end of treatments, but did not receive the second-line therapy. Cost of the final month of life was assigned at death. Monthly monitoring, postprogression, and end-of-life costs are summarized in the Data Supplement. All costs were adjusted to 2021 USD using the Personal Consumption Expenditures price index for health care services.

### Sensitivity Analyses

Point estimates for each input parameter were used in the primary analysis. We also performed one-way sensitivity analyses on the cost of medication to identify the threshold at which more costly treatments would become cost-effective at a willingness-to-pay (WTP) threshold of $150,000 USD/QALY. Deterministic sensitivity analyses on drug and ADE treatment costs and utilities with 20% variation (tornado analysis) were performed to identify high-impact parameters in the model. We did not conduct probabilistic sensitivity analyses because of the lack of data regarding the variation of input parameters.

## RESULTS

The results from the primary analysis showed that 10-year total costs—inclusive of the medications, their administration, and ADEs—were the lowest for sunitinib ($467,394 USD-$726,068 USD) and highest for either LP or PA ($1,116,302 USD-$1,502,129 USD), regardless of the perspective and the rate of subsequent therapy (Table 1). Estimated 10-year QALYs ranged from 2.52 for AA to 3.60 for NI. AB, AA, NC, LP, and PA were eliminated from consideration because they were less effective and more costly than sunitinib or NI (dominance) or a linear combination of the two (extended dominance). The incremental cost-effectiveness ratio for NI, compared with sunitinib, varied from $297,465 USD/QALY (VA perspective and 30% of patients receiving second-line therapy) to $422,220 USD/QALY (Medicare/Medicaid perspective and 100% of patients receiving second-line therapy), depending on the study perspectives and the rate of subsequent therapy (Table 1).

**TABLE 1.**
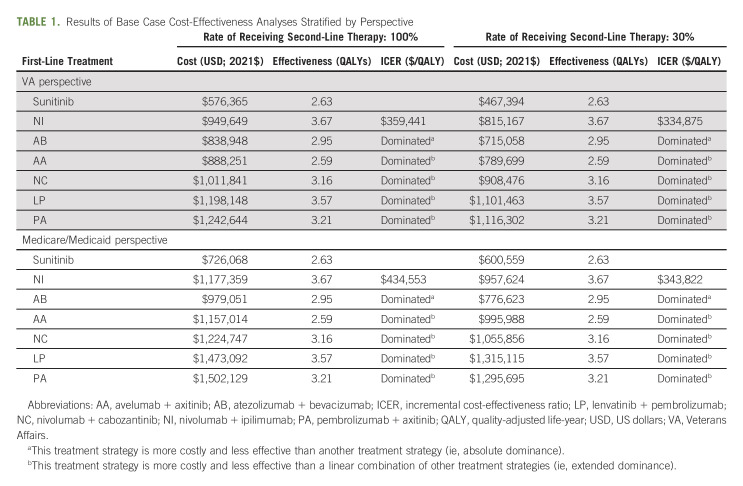
Results of Base Case Cost-Effectiveness Analyses Stratified by Perspective

**FIG 2.**
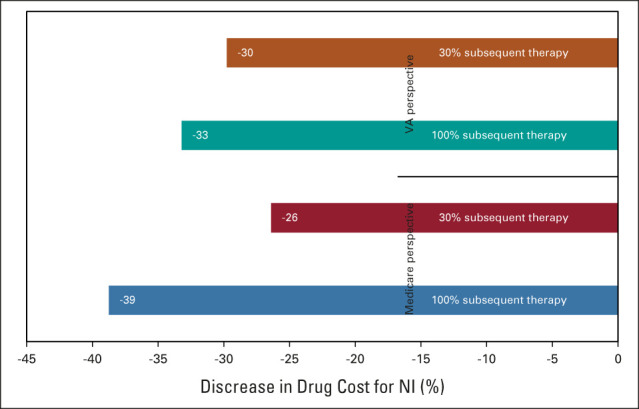
Results of univariable sensitivity analysis on drug cost for NI: % decrease required for NI to be cost-effective relative to sunitinib at a willingness-to-pay threshold of $150,000 US dollars/quality-adjusted life-year. NI, nivolumab + ipilimumab; VA, Veterans Affairs.

The results of the one-way sensitivity analysis on the direct cost of NI are illustrated in Figure 2. This analysis showed that the incremental cost-effectiveness ratio for NI relative to sunitinib falls below $150,000 USD/QALY if the direct cost of NI for the first four months decreases by 26% to 39%, depending on the scenario. The tornado analysis suggested that the utility for NI and S had the greatest impact on the results (Data Supplement).

## DISCUSSION

mRCC management has seen a rapid evolution of the therapeutic landscape since 2017 with the introduction of several novel immune checkpoint inhibitors and targeted therapy combinations of both classes of drug agents. Immunotherapeutic-based drug combinations that have emerged as first-line choices have increased response rates and longevity of life and provided a fundamental shift from interferon and interleukin-based interventions. However, these contemporary therapies lack validated predictive biomarkers to guide choices in individual patients, and although response rates have increased with these combinations, not all patients have clinical benefit and all drug regimens are associated with significant side effects and financial toxicity. In fact, higher drug costs have been widely reported across the spectrum of cancer drug treatments and the majority of newly approved anticancer molecular entities lack validated cancer biomarkers for identifying patient subcohorts most likely to benefit within the same tumor type.^25^ In the absence of validated companion biomarker-based guidance for individual approved therapeutic combinations, the determination of cost-effectiveness has become an important tool to pursue for individualizing drug treatment choices. We attempted to evaluate comparative costs and clinical impact of all approved standard-of-care drug combinations in the first-line setting used currently for treating mRCC. We used published randomized clinical trial survival and adverse effects data and estimated cost-effectiveness for a 10-year horizon. We also assumed two different clinical scenarios of therapeutic interventions in the second-line treatment setting. These basic assumptions are in line with currently accepted clinical practice and the methods used for economic evaluations in oncology^12^ including the preferred use of PSA in cost evaluation strategies when evaluating novel IO-based drug therapies.^26,27^

Our results demonstrate that sunitinib was the most cost-effective first-line strategy at a WTP of $150,000 USD/QALY, which is the threshold typically used by the US Institute for Clinical and Economic Research^28^ because of lower drug-associated costs compared with all other approved IO-IO and IO-TKI combinations. Among combination therapies, NI was the only strategy not dominated by sunitinib. NI was associated with higher QALYs over a 10-year time horizon versus all other options including sunitinib. With higher WTP thresholds, NI was observed to be the most cost-effective treatment strategy. The results were consistent whether we used VA FSS or ASP/NADAC drug costs or whether our model assumed that only some or all patients received second-line therapy after failure of first-line treatments.

Our results differ from recently published cost-effective studies for mRCC on the basis of study design and analyses. For instance, Shay et al^15^ found that NI was cost-effective compared with PA and AA; however, in the study, the authors did not include sunitinib as a comparator. Zhu et al^29^ and Lu et al^30^ did not find PA or AA to be cost-effective relative to sunitinib. In several studies, PA was reported as being cost-effective compared with NI and/or sunitinib^14,20,31,32^ and NI was reported as cost-effective as sunitinib.^13^ A key difference between our analyses and recent publications was the assessment of drug costs. Many of these recent published studies used the drug manufacturer's list price or wholesale acquisition cost, which does not reflect the actual cost paid for drugs because it does not include discounts and rebates. For this reason, Levy et al^33^ proposed to use either the NADAC and VA Federal Supply Schedule Service or a combination of the two, which we used to reflect the drug supply system. Another major factor that could explain the difference between our findings and those from previous studies is the rate of subsequent therapy. We ran a version of our model in which all patients received second-line therapy and then another with only 30%. Other studies mostly applied around 30% of subsequent therapy on the basis of the reports from clinical trial, which might not reflect the actual clinical practice as second-line treatments are now well embedded in clinical practice. In addition, although there have been many published CEAs already of first-line treatments in mRCC, our study is comprehensive in that it considers all seven first-line approved treatments, whereas others have included a subset of these therapies.

Our study is not without limitations. First, uncertainty is always present when extrapolating survival data to a 10-year time horizon. To reduce this uncertainty, we tested various potential parametric survival functions to find the distribution that most accurately captures the unique characteristics of data in our study. Second, to compare seven different treatments, we used six independent clinical trials that compared each IO-TKI combination with sunitinib. The Kaplan-Meier PFS and OS curves for sunitinib vary across each of these studies. To incorporate this variation into our analysis, we ran our model separately using both the highest and the lowest reported median time for PFS across these six trials. Third, we focused our analysis for the overall patient population from these trials. We did not conduct subgroup analyses in patients from different prognostic groups because a priori enrollment of patients in these prognostic group was not reported consistently in the six clinical trials. Fourth, although the drug cost estimates from VA Federal Supply Schedule Service or ASP/NADAC represent the actual drug costs better than the wholesale acquisition cost, there still exists a large variation in drug costs for different payers across the United States. For example, costs from the private payer perspective were not considered in our study. In addition, we did not include patient out-of-pocket costs, but they may be important as evidenced in previous studies.^34^ Finally, we did not conduct probabilistic sensitivity analyses because we did not have information on the distribution of most input parameters. Adding this would provide more information as to the consistency of our results across a wide range of potential values for input parameters.

In conclusion, using a partitioned survival model parameterized using inputs from recent clinical trials, we found that NI yielded the most QALYs of the seven treatments considered. However, despite being the most effective first-line strategy, the treatment, administration, and ADE costs of NI were high enough that sunitinib was the most cost-effective treatment strategy in our model at a WTP of $150,000 USD/QALY.
